# GBR 12909, a Selective Dopamine Transporter Inhibitor, Differentially Modulates Affective, Exploratory and Cognitive Domains in Zebrafish

**DOI:** 10.1111/ejn.70651

**Published:** 2026-08-19

**Authors:** Camilla W. Pretzel, Hevelyn S. Moraes, Mariana L. Müller, Kimberly Fontoura, Rossano S. Menezes, Julia Canzian, Rodrigo E. Barreto, Cassio M. Resmim, Barbara D. Fontana, Denis B. Rosemberg

**Affiliations:** ^1^ Laboratory of Experimental Neuropsychobiology, Department of Biochemistry and Molecular Biology, Natural and Exact Sciences Center Federal University of Santa Maria Santa Maria Rio Grande do Sul Brazil; ^2^ Graduate Program in Biological Sciences: Toxicological Biochemistry Federal University of Santa Maria Santa Maria Rio Grande do Sul Brazil; ^3^ Department of Biochemistry and Molecular Biology, Natural and Exact Sciences Center Federal University of Santa Maria Santa Maria Rio Grande do Sul Brazil; ^4^ Department of Structural and Functional Biology (Physiology) Institute of Biosciences of Botucatu, UNESP Botucatu Brazil; ^5^ The International Zebrafish Neuroscience Research Consortium (ZNRC) Slidell Louisiana USA

**Keywords:** affective‐like behaviour, cognition, dopamine transporter inhibition, exploratory behaviour, working memory

## Abstract

Dopamine plays an important role in several biological functions, such as reward, movement regulation and cognition. Physiological alterations in dopamine transporter (DAT) are related to psychiatric conditions, including bipolar disorder (BD), a condition characterized by intense shifts in mood, behaviour and energy, alternating between manic and depressive states. Administration of GBR 12909, a selective dopamine reuptake inhibitor, has been considered a suitable model to assess BD‐related phenotypes, yet the integration of anxiety‐like, despair‐like, cognitive and spatiotemporal behavioural dimensions has not been systematically evaluated. Here, we evaluated the acute behavioural effects of GBR 12909 using a multidomain behavioural assessment in adult zebrafish. Fish received a single intraperitoneal administration of GBR 12909 (15 mg/kg) and 30 min after, were challenged to the open field test (OFT), light–dark test (LDT), shallow water test (SWT) and Free Movement Pattern Y‐Maze (FMP Y‐Maze). GBR 12909‐treated zebrafish exhibited increased time in homebase during OFT, prominent scototaxis and hyperactivity in LDT, increased immobility and hypolocomotion in SWT, along impaired cognitive flexibility and stereotypical behaviours in the FMP Y‐maze. These data reflect affective and cognitive disturbances consistent with dopaminergic overstimulation that triggers manic‐ and depressive‐like phenotypes, thereby recapitulating behavioural phenotypes relevant to multiple domains affected in BD. Collectively, our findings support that GBR12909 elicits dopaminergic‐driven behavioural phenotypes relevant to multiple domains of BD, providing a translational model for studying the neurobiological bases of DAT inhibition across distinct behavioural domains.

## Graphical abstract

Dopamine transporter inhibition is linked to bipolar disorder, a cyclic condition difficult to mimic in animal models. Here we observed that a single dose of GBR12909 disrupted zebrafish behaviour across multiple domains, triggering anxiety‐ and despair‐like states alongside impaired working memory and cognitive flexibility. This multidomain profile reflects core features of bipolar disorder, highlighting zebrafish as a powerful translational model for dopaminergic neuropsychiatric conditions.

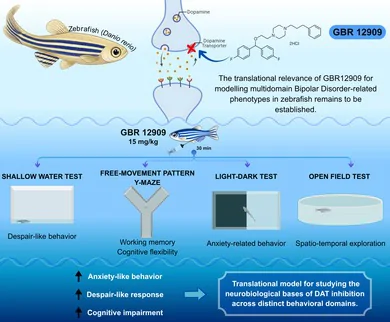

**Abbreviations**
BD: bipolar disorder
DAT: dopamine transporter
FMP Y‐MAZE: Free Movement Pattern Y‐maze
HI: homebase index
LDT: light–dark test
OFT: open field test
RM‐ANOVA: repeated measures two‐way analysis of variance
SEM: standard error of the mean
SWT: shallow water test

## 1 Introduction

Dopamine is an important neurotransmitter involved in the modulation of distinct behavioural domains, especially reward, movement regulation and cognitive processes (Bissonette and Roesch 2016; Costa et al. 2014; Juárez Olguín et al. 2016). In the brain, dopamine levels are regulated by dopamine transporter (DAT) (Nieoullon 2002; Westbrook and Braver 2016; Liu and Kaeser 2019; Meder et al. 2019). DAT is an integral membrane protein that functionality regulates dopaminergic neurotransmission, controlling the dopamine availability in the synaptic cleft, which may affect distinct behavioural outcomes (Bu et al. 2021; Nepal et al. 2023). Importantly, DAT is expressed not only in neurons but also in astrocytes, linking dopamine‐dependent mechanisms throughout the striatum and contributing to the maintenance of dopamine homeostasis (Hynes et al. 2024). Dysregulation of dopamine reuptake due to altered DAT function has been implicated in the pathophysiology of several psychiatric disorders, such as attention‐deficit/hyperactivity disorder, obsessive‐compulsive disorder, schizophrenia and bipolar disorder (BD) (Horschitz et al. 2005; Vaughan and Foster 2013; Herborg et al. 2018).

BD is a mental condition that affects approximately 39.5 million people worldwide (Jiang et al. 2025; Liu et al. 2025). Despite the biological basis of BD remains to be fully elucidated, the aetiology of disorder involves a complex interaction of genetic, neurochemical and environmental factors (Harrison et al. 2018; Jain and Mitra 2025). The main symptoms of BD are episodes of mania or hypomania alternating with depression. In mania phases, BD patients often experience elevated mood and energy, distractibility, hallucinations, hyperactivity, increased talkativeness, rapid speech, racing thoughts, increased goal‐directed activity and psychomotor agitation (Benazzi and Akiskal 2006; Dailey and Saadabadi 2025). Conversely the depressive phase of BD is characterized by sadness, hopelessness or worthlessness accompanying low energy levels and constant fatigue (Jain and Mitra 2025). In addition to mood alterations, individuals with BD frequently exhibit exacerbated anxiety and cognitive impairments, including attention deficits, executive function, learning, memory and cognitive flexibility, which substantially contribute to functional disability and poor clinical outcomes (Harrison et al. 2018; Spoorthy et al. 2019; Huang et al. 2023; Jain and Mitra 2025; Steardo et al. 2025).

BD has been associated with dysregulation in dopaminergic neurotransmission (Bissonette and Roesch 2016; Ashok et al. 2017). During mania/hypomania, a hyperdopaminergic is observed, together with high availability of D2 and D3 striatal receptors and hyperactivity in reward‐processing networks (Berk et al. 2007; Ashok et al. 2017). The depressive phase is associated with hypodopaminergia, due to elevated DAT levels and decrease in D1‐like receptors potentially leading to reduced dopaminergic function (Suhara et al. 1992; Berk et al. 2007; Ashok et al. 2017; Scaini et al. 2020). This imbalance suggests a failure in the homeostasis between dopamine receptors and transporters (Ashok et al. 2017; Scaini et al. 2020). Because BD encompasses both manic and depressive symptoms, associated with multiple underlying mechanisms, it remains difficult to treat with a single therapeutic approach. Consequently, patients frequently experience persistent residual symptoms, including psychosocial dysfunction and cognitive impairment (Bonnín et al. 2019; Grunze and Born 2020).

Although rodent models have traditionally dominated BD research, recently, alternative vertebrate models have increasingly been employed, complementing the existing murine approaches (Bonan and da Silva Carlini 2022; Canzian et al. 2022; Freund and Juckel 2019; Logan and McClung 2016). The zebrafish ( *Danio rerio* ) is a popular model organism in neuropsychiatric research, due to the high genetic similarity and the conservation of neurochemical, molecular, endocrine and physiological systems relative to mammals, including the dopaminergic system (Howe et al. 2013; Khan et al. 2017; Gerlai 2020; Pansera et al. 2025). Zebrafish also display a rich, well‐characterized and highly reproducible behavioural repertoire (Fontana et al. 2018; Kalueff et al. 2025, 2013), supporting the utility of this species to investigate how changes in dopaminergic neurophysiology are involved in multiple symptoms associated with brain disorders (Boehmler et al. 2004; Ek et al. 2016; Wasel and Freeman 2020; Canzian et al. 2022, 2025).

Evidence shows the pharmacological use of GBR 12909 as a selective dopamine uptake inhibitor (Andersen 1989). GBR 12909 has been used in both rodents and zebrafish for modelling mania‐like behaviour, serving as a useful tool to assess the neurobiological mechanisms and potential therapeutic interventions for BD (Bigot et al. 2022; Canzian et al. 2024, 2025). Similarly to other DAT inhibitors and psychostimulants drugs, GBR 12909 elicits hyperactivity and oxidative imbalance in rodents that mimic BD‐related symptoms (Young et al. 2010; Queiroz et al. 2015; Bastos et al. 2018). In zebrafish, the effects of GBR 12909 have previously been assessed in the novel tank test, where hyperactivity and alterations in redox homeostasis were observed (Canzian et al. 2024). However, it remains unclear whether GBR 12909 can affect a broader multidomain behavioural phenotype of BD, including cognitive dysfunction, anxiety‐related alterations and depressive‐like features, constituting a key factor for improving translational interpretability. Thus, our goal was to evaluate the influence of GBR 12909 on different behavioural domains in adult zebrafish. For this, four experiments were conducted: the open field test (OFT), commonly used to assess the overall spatiotemporal exploratory activity (Stewart et al. 2010, 2012; Resmim et al. 2023; Borba et al. 2025); the light–dark preference test (LDT), to quantify anxiety‐related behaviours (Maximino et al. 2011); the shallow water test (SWT), which provides a robust framework for assessing depressive‐like behaviour through the evaluation of behavioural despair (Müller et al. 2025); and the free‐movement pattern (FMP) Y‐maze test, which provides a tool to quantify changes in cognitive flexibility, working memory and repetitive behaviours (Cleal, Fontana, Ranson, et al. 2021). Thus, considering the multiple behavioural phenotypes associated with BD and the central role of dopamine in locomotion, emotional regulation, reward processing, and cognition, we hypothesized that acute GBR 12909 administration would disrupt multiple behavioural domains. Specifically, we expected GBR 12909 to alter exploration in the OFT (i.e., by changing homebase‐related activity), trigger anxiety‐related responses in the LDT (i.e., by increasing dark preference), elicit despair‐like behaviour in the SWT (i.e., by increasing immobility) and impair cognitive performance in the FMP Y‐maze, particularly cognitive flexibility and working memory, domains consistently affected in patients with BD. Together, these assays were designed to characterize the multidomain behavioural consequences of acute DAT blockade and to evaluate their translational relevance to behavioural dimensions associated with BD.

## 2 Material and Methods

### 2.1 Animals

Adult zebrafish (4–6 months old, ~50:50, male:female ratio, weighing ~0.5 g, short fin phenotype) were obtained from a commercial supplier (Hobby Aquários, RS, Brazil). Fish were maintained in 40‐L tanks filled with dechlorinated water under constant mechanical and chemical filtration for at least 14 days before the experiments (stocking density of two fish per litre). Animals were kept under standard water conditions (temperature at 27°C ± 1°C, pH 7.0–7.2, conductivity at 600–800 μS/cm, dissolved oxygen at 6.0 ± 0.1 mg/L, total ammonia at < 0.1 mg/L, alkalinity and hardness at 75 mg/L CaCO_3_), with illumination provided by fluorescent lamps on a 14/10‐h light/dark photoperiod cycle (lights on at 7:00 AM and off at 9:00 PM) (Avdesh et al. 2012; Aleström et al. 2020). The subjects used were experimentally naive and fed three times per day with commercial flake fish food (Tropical SUPERVIT, TROPICAL, Brazil). All procedures were carried out following the National Institute of Health Guide for Care and Use of Laboratory Animals, and the experimental protocols were approved by the Institutional Animal Care and Use Committee (Process Number 9102110723).

### 2.2 Experimental Procedures

**FIGURE 1:**
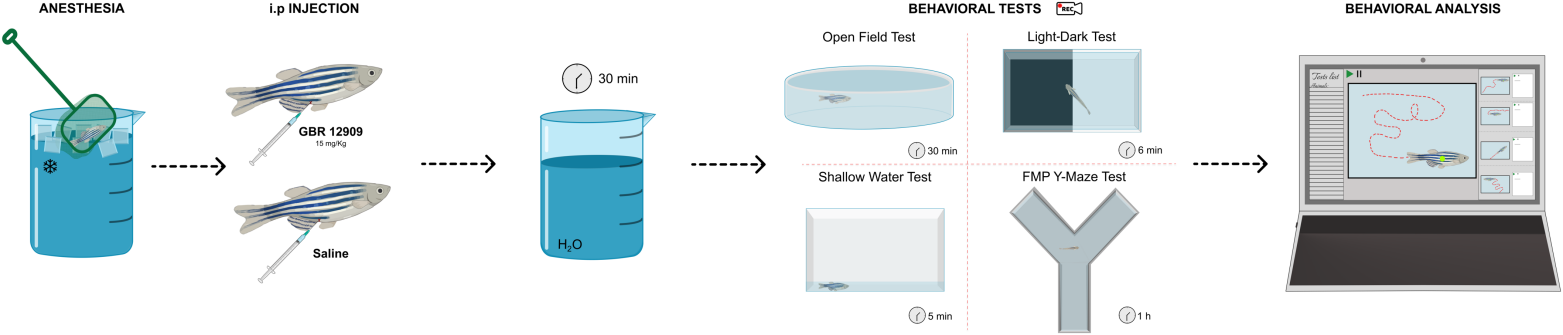
Schematic representation of the experimental design. Adult zebrafish were first weighed and isolated in acclimatation tanks for at least 24 h. For the experimental procedure, each fish was anaesthetized using cold water at 4°C and injected with saline or GBR12909 and placed in a beaker with 500 mL of water dechlorinated for 30 min. Immediately after this period, animals were tested in the open field test for 30 min or in the Light–dark test and shallow water test for 6 and 5 min, respectively, or in the FMP Y‐Maze test for 1 h. Data were further analysed offline by an automated video‐tracking system.

All the experiments were conducted between 8:00 AM and 5:00 PM, in a randomized treatment order and testing to ensure data replicability. Behavioural assays were recorded with the camera positioned 40 cm above the water surface, with light intensity maintained at 180 lx at the water level to ensure consistent and uniform illumination and reliable fish detection. Different cohorts of animals were used for each behavioural assay. An exception was made for the LDT and SWT, in which the same animals were sequentially evaluated. The LDT was conducted prior to the SWT, as both procedures are brief and the testing sequence progressed from a less aversive to a more aversive paradigm (Serchov et al. 2016; Fontana et al. 2022; Müller et al. 2025). This approach was implemented to reduce the total number of animals used, in accordance with the principles of the 3Rs (replacement, reduction and refinement). Moreover, control and GBR12909‐treated animals were assessed under identical experimental conditions and in parallel sessions to avoid environmental bias. The tank water was changed after each trial to ensure adequate water conditions and avoid a potential influence of the previous animal. Videos were recorded at 30 frames/s and further analysed offline with the ANY‐maze software (Stoelting, CO, USA) to eliminate observer bias with all data extracted automatically. In the end of the behavioural experiments, each animal was anaesthetised in cold water and euthanatized. Figure 1 shows a schematic representation of the experimental protocols used in this study.

### 2.3 GBR 12909 Preparation and Administration

GBR 12909 dihydrochloride (Sigma‐Aldrich, St. Louis, MO, USA) was solubilized in saline and sonicated for 45 min at 28°C before use (Canzian et al. 2024). The dilution for injections was performed from a stock solution of 1.5 mg/mL. Fish were weighed and isolated in an acclimatation tank for at least 24 h to minimize isolation stress (Franscescon et al. 2020). The acclimatation tank consists of a rectangular apparatus (75‐cm length × 30‐cm width × 10‐cm height) with 16 chambers (9‐cm × 9.5‐cm × 10‐cm length, width and height, respectively) with 2‐mm perforations to allow appropriate water circulation and aeration (Resmim et al. 2025). After the acclimation period, animals were gently anaesthetised in water at 4°C and briefly immobilized using a small wet fishing net (Kinkel et al. 2010; Canzian et al. 2024). Animals were then injected intraperitoneally with saline (vehicle) or GBR 12909 (15 mg/kg) into the midline between the pelvic fins, through the net. All injections were performed using a BD Ultra‐fine 30 U syringe (needle size 6 mm × 0.25 mm) with a volume of 10 μL. The dose and the protocol chosen were based on previous reports that assessed BD‐like responses in zebrafish, which induced hyperlocomotion, anxiety‐ and depression‐like responses, as well as decreased social preference (Canzian et al. 2024).

### 2.4 Behavioural Tests

#### 2.4.1 OFT

To assess the overall exploratory profile, zebrafish were subjected to the OFT (Kraeuter et al. 2019; Borba et al. 2023). Behaviours were assessed in a circular‐shaped arena, with transparent walls and a bright background, with dimensions of 25 × 6 cm (diameter × water column height). The apparatus was virtually divided in 16 sections (eight in centre, eight in periphery), periphery was set at 6 cm from the edge. Fish (*n* = 12 per group) were gentle placed in the centre of the arena and locomotion‐, exploration‐, and homebase‐related behaviours were measured in a 30‐min trial. Data were then exported separately into Excel spreadsheets for further analyses, in which the identification of the homebase section was based on previous studies (Borba et al. 2022; Resmim et al. 2023; Pretzel et al. 2024). We estimated the homebase index (HI) by multiplying the results of distance travelled × time spent × the number of entries in each zone to estimate the region of the apparatus with pronounced activity. The area with the highest HI value for each fish was defined as the homebase. To analyse exploratory excursions (i.e., exploration starting from the homebase section of each animal), we calculated the number of trips; average length of trips (distance travelled in non‐homebase areas divided by the transitions to the homebase); average duration of trips (determined as the ratio of time spent in non‐homebase areas and transitions to the homebase); and the average number of stops per trip (calculated by dividing the immobile episodes in non‐homebase areas by the number of trips) (Borba et al. 2022, 2023; Resmim et al. 2023; Pretzel et al. 2024).

#### 2.4.2 LDT

To assess anxiety‐related behaviours, zebrafish were tested in LDT as described previously (Maximino et al. 2011; Mezzomo et al. 2016; Canzian et al. 2019). Each fish (*n* = 12 per group) was individually tested in a rectangular glass tank (25‐cm length × 15‐cm height × 11‐cm width), divided equally into two sections. The walls and floor were externally covered with black and white self‐adhesive film to create a contrast between both sections. Animals were gently placed in the light area and behaviour was recorded for 6 min. The following parameters were quantified: number of transitions between compartments, time spent in the lit area, and latency to enter the dark area (Kalueff et al. 2025).

#### 2.4.3 SWT

The SWT was employed to measure despair‐like behaviours (Müller et al. 2025). Behavioural despair reflects intense hopelessness, a key symptom of depression and other psychiatric disorders (Goodwin and Stein 2021; Kositsyn et al. 2022). In the SWT, despair‐like behaviour is inferred from diminished escape activity in an aversive context, reflected by increased immobility and hypolocomotion (Müller et al. 2025). Immediately after the LDT, the same animals (*n* = 12 per group) were individually transferred to a rectangular tank (15‐cm length × 10‐cm width × 13‐cm height) covered externally with white self‐adhesive film. The shallow environment was set up with 120 mL of dechlorinated providing a water column height of approximately 1 cm in which zebrafish can swim freely while having the dorsal fin exposed. Behaviours were recorded for 5 min and the endpoints were analysed: distance travelled, average velocity, maximum speed, absolute turn angle, immobility duration and latency to mobility (Müller et al. 2025).

#### 2.4.4 FMP Y‐Maze Test

To evaluate memory and cognitive parameters in zebrafish, the FMP Y‐maze task was performed based on previous reports (Cleal, Fontana, Double, et al. 2021; Cleal, Fontana, Ranson, et al. 2021; Fontana et al. 2025). In this test, we assess the right and left turn choices during free exploration in an apparatus with absence of visual cues, relating with working memory and cognitive flexibility (Cleal, Fontana, Ranson, et al. 2021; Fontana et al. 2025). In general, vertebrates adopt exploratory strategies based on directional alternation, characterized by sequences such as left–right–left–right (LRLR) or right–left–right–left (RLRL), reflecting a natural predisposition to this behavioural pattern, while repetitive behaviours (RRRR or LLLL) are usually induced by abnormal states such drugs that induce stereotypical behaviours and acute stressors, which can be associated with impairment in working memory (Cleal, Fontana, and Parker 2021; Cleal, Fontana, Ranson, et al. 2021; Fontana et al. 2021). The apparatus consisted of a Y‐maze tank with three arms (5 cm long, 2 cm wide and 14 cm high), externally covered with white self‐adhesive film, and filled with 350 mL of dechlorinated water (10‐cm water column height). Fish (*n* = 14 per group) were individually transferred to the middle of the three arms and behaviour was monitored for 1 h. The following parameters were evaluated: number of turns, left turns, right turns, behavioural tetragrams, alternations across time, total alternations, repetitions across time and total repetitions. To assess the behavioural tetragrams, zebrafish behaviour was categorized into all possible turn patterns (LLLL, LLLR, LLRL, LLRR, LRLL, LRLR, LRRL, LRRR, RLLL, RLLR, RLRL, RLRR, RRLL, RRLR, RRRL, RRRR), with R indicating the fish turning to the right and L to the left. Alternations were considered as RLRL or LRLR and repetitions as LLLL or RRRR (Fontana et al. 2025).

### 2.5 Statistics

Data normality and variance homogeneity were assessed using the Kolmogorov–Smirnov and Brown–Forsythe's tests, respectively. Results were expressed as mean ± standard error of the mean (SEM) and nonparametric data were log‐transformed. Statistical comparisons were performed using the unpaired Student's *t*‐test or repeated measures two‐way analysis of variance (RM‐ANOVA) depending on the experimental design. For the tetragram analysis, a two‐way repeated‐measures ANOVA with Geisser–Greenhouse correction was used. Individual data points were shown to facilitate visualization of data distribution. Post hoc analyses were carried out by Tukey's multiple comparisons test whenever appropriate. Although number of subjects for measuring the behavioural responses were based on previous studies using adult zebrafish (Canzian et al. 2024; Pretzel et al. 2024; Fontana et al. 2025; Müller et al. 2025), the minimum sample size required for the experiments (*n* = 12) was also confirmed using G‐Power 3.1.9.7 for Windows for a two‐group comparison with an effect of 1.4 and *α* = 0.05, indicating that the number of fish used would provide adequate statistical power. Statistics were performed by the GraphPad Prism 8.0 software and results were considered significant when *p* ≤ 0.05. Additionally, for the analysis of the FMP Y‐maze test, RStudio (R Version 4.3.2) was used to process and compile the data for tetragram quantification, across‐time analysis, alternations, and repetitions. The total recording period was divided into six consecutive 10‐min trials to reconstruct the turning sequence of each animal within each trial. The raw entrance in each arm of the maze was exported from ANY‐maze and processed in RStudio, where consecutive arm transitions were converted into relative left (L) and right (R) turn choices based on the geometry of the Y‐maze. These binary turn sequences were then organized into tetragrams consisting of four consecutive turn choices. Since four binary turn choices generate 16 possible tetragram sequences (e.g., RRRR, RRRL and RLRL), this approach enabled the systematic classification and quantification of each tetragram pattern for subsequent behavioural analysis. Importantly, two trained observers blinded to the experimental conditions analysed the results independently. All researchers involved in the animal handling, recording and data analysis were blinded to treatment allocation. Only one researcher who administered the treatments knew the treatment allocation and was not involved in any experimental or analytical step. The treatment allocation was revealed only after all analysis had been completed.

## 3 Results

### 3.1 GBR 12909 Modulates Homebase Occupancy in the OFT

**FIGURE 2:**
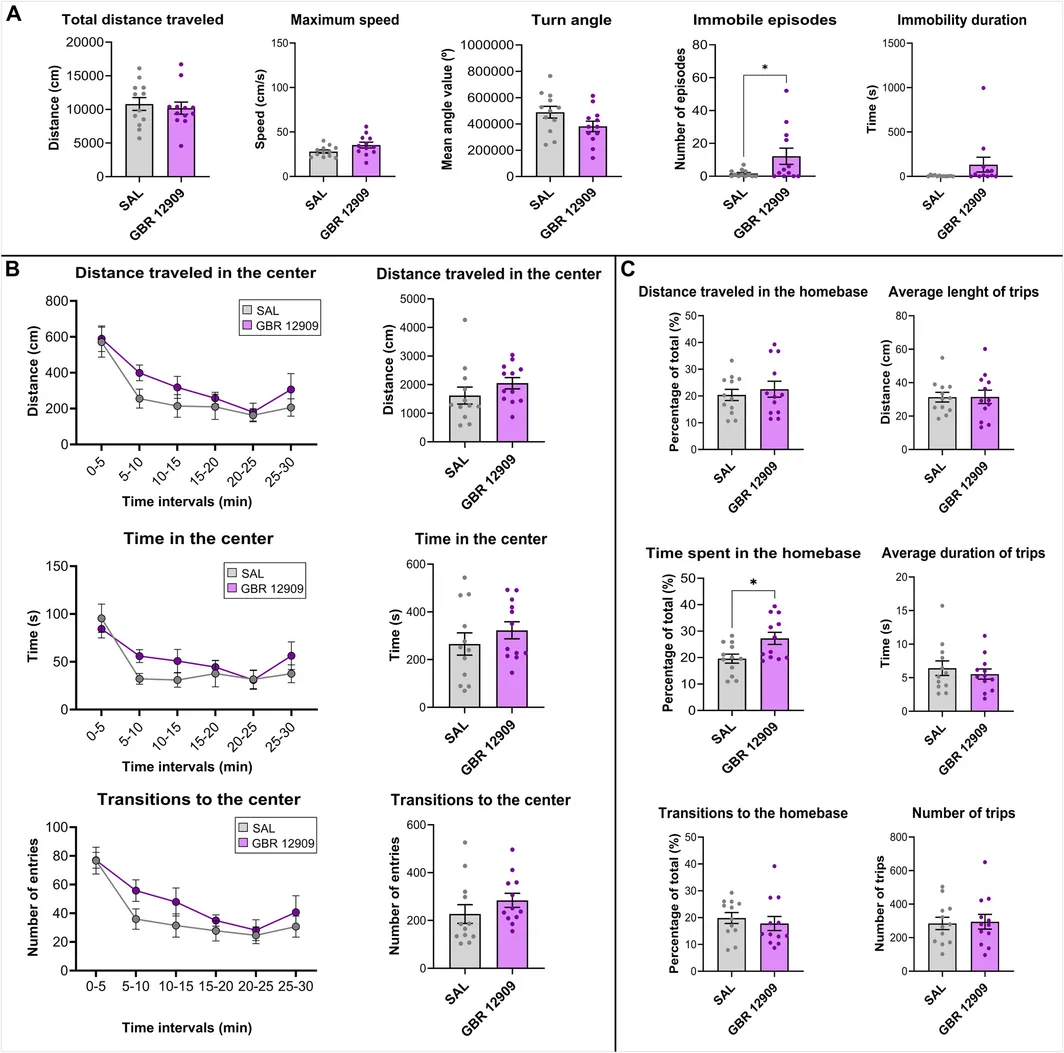
Influence of GBR 12909 on spatio‐temporal exploratory dynamics in the open field test. (A) Overall locomotor parameters. (B) Exploration of the centre area parameters as endpoints and across time. Temporal analysis of behaviour was calculated as the area under the curve (AUC) for each experimental subject in 5‐min time intervals. (C) Homebase‐related parameters. Data are expressed as means ± S.E.M. and analysed by Student's *t*‐test or two‐way RM‐ANOVA, depending on the experimental approach. Post hoc analysis was carried out by the Tukey's multiple comparisons test (*p ≤ 0.05, **p ≤ 0.01, ***p ≤ 0.001, ****p ≤ 0.0001; *n* = 12 per group).

To elucidate the role of GBR 12909 in the OFT (Figure 2), we first assessed the effects on locomotion‐based endpoints (Figure 2A). GBR 12909 group showed an increased number of immobile episodes (*t*_*(22)*_ = 2.120, *p* = 0.0455, *d* = 0.9039). However, no differences were found for total distance travelled (*t*_*(22)*_ = 0.4732, *p =* 0.6407, *d* = 0.2017), maximum velocity (*t*_*(22)*_ = 1.977, *p* = 0.0607, *d* = 0.8429), absolute turn angle (*t*_*(22)*_ = 1.814, *p =* 0.0834, *d* = 0.7734) and immobility duration (*t*_*(22)*_ = 1.534, *p* = 0.1394, *d* = 0.6540). Concerning the occupation pattern (Figure 2B), the distance travelled in the centre (*t*_*(22)*_ = 1.214, *p =* 0.2376, *d* = 0.5176), transitions to the centre (*t*_*(22)*_ = 1.161, *p* = 0.2580, *d* = 0.4950) and time in the centre (*t*_*(22)*_ = 0.9730, *p* = 0.3411, *d* = 0.4148) did not change. Temporal analysis of behaviour yielded significant effects of time for distance travelled in the centre (*F*_(5, 110)_ = 17.50, *p* < 0.0001, *ηp* ^2^ = 0.4430), transitions to the centre (*F*_(5, 110)_ = 20.07, *p* < 0.0001, *ηp* ^2^ = 0.4770) and time in the centre (*F*_(5, 110)_ = 11.74, *p* < 0.0001, *ηp* ^2^ = 0.3479), while no effects on time × treatment interaction or treatment were observed. Overall, for both groups the activity in the centre zone was decreased over time compared with the first 5 min. For homebase occupancy (Figure 2C), we did not find significant effects for distance travelled in the homebase (*t*_*(22)*_ = 0.5923, *p* = 0.5597, *d* = 0.2525) and transitions to the homebase (*t*_*(22)*_ = 0.6131, *p* = 0.546, *d* = 0.2614). However, GBR 12909‐treated fish significantly spent more time in the homebase (*t*_*(22)*_ = 2.686, *p* = 0.0135, *d* = 1.453). Analysis of the exploratory activity from the homebase section revealed no effects in the number of trips (*t*_*(22)*_ = 0.1764, *p* = 0.8616, *d* = 0.0752) (data not shown), average length of trips (*t*_*(22)*_ = 0.02868, *p* = 0.9774, *d* = 0.0122), average duration of trips (*t*_*(22)*_ = 0.6664, *p* = 0.5121, *d* = 0.2841) and number of stops per trip (*t*_*(22)*_ = 1.631, *p* = 0.1172, *d* = 0.6954).

### 3.2 GBR 12909 Elicits Anxiety‐Like Responses in the LDT and Despair‐Like Behaviours in SWT

**FIGURE 3:**
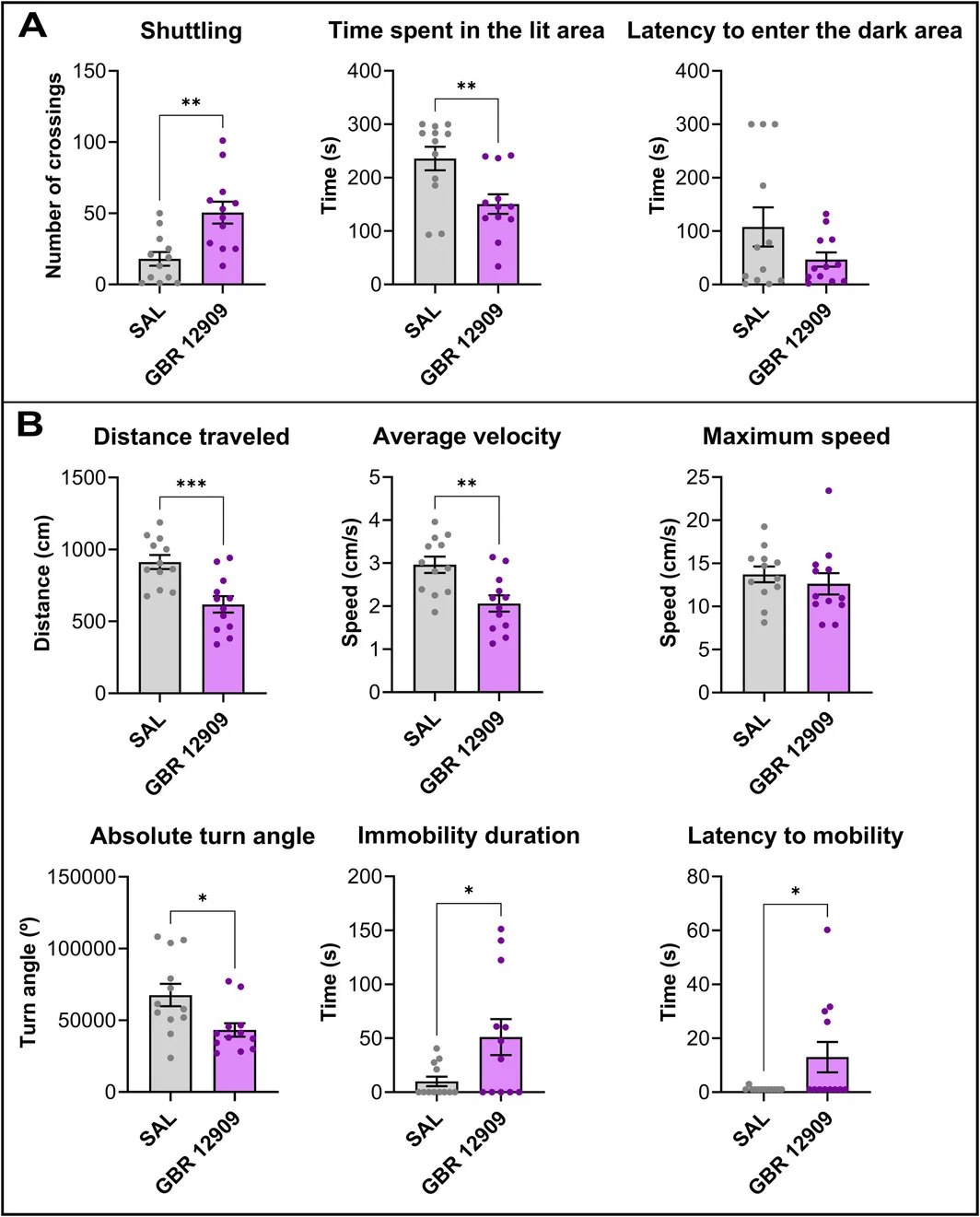
Effects of GBR 12909 on affective‐like behaviours. (A) Anxiety‐like behaviours measured with light–dark test. (B) Despair‐like behaviours assessed in shallow water test. Data are expressed as means ± S.E.M and analysed by Student's *t*‐test (*p ≤ 0.05, **p ≤ 0.01, ***p ≤ 0.001, ****p ≤ 0.0001; *n* = 12 per group).

Figure 3A shows the effects of GBR 12909 on anxiety‐like responses measured in the LDT. Here, we found that GBR 12909‐exposed fish had increased shuttling (*t*_*(22)*_ = 3.572, *p* = 0.0017, *d* = 1.5231) and spent less time in the lit area (*t*_*(22)*_ = 2.968, *p* = 0.0071, *d* = 1.2655), while the latency to enter the dark area did not differ between groups (*t*_*(22)*_ = 1.510, *p* = 0.1307, *d* = 0.6438). Meanwhile, in the SWT, locomotion‐ and immobility‐related parameters were used to assess specific phenotypes resembling behavioural despair and anxiety (Figure 3B). GBR 12909 markedly reduced general locomotion‐based behaviours, such as distance travelled (*t*_*(22)*_ = 3.950, *p* = 0.0007, *d* = 1.6842), average velocity (*t*_*(22)*_ = 3.367, *p* = 0.0028, *d* = 1.4356) and absolute turn angle (*t*_*(22)*_ = 2.668, *p* = 0.0140, *d* = 1.1376) when compared to the saline (control) group, while no changes were found in the maximum speed (*t*_*(22)*_ = 0.7127, *p* = 0.3213, *d =* 0.3038). Moreover, both immobility duration (*t*_*(22)*_ = 2.379, *p* = 0.0265, *d* = 1.0144) and latency to mobility (*t*_*(22)*_ = 2.099, *p* = 0.0475, *d* = 0.8950) were increased in GBR 12909 group.

### 3.3 GBR 12909 Impairs Working Memory and Reduces Cognitive Flexibility in the FMP Y‐Maze Test

**FIGURE 4:**
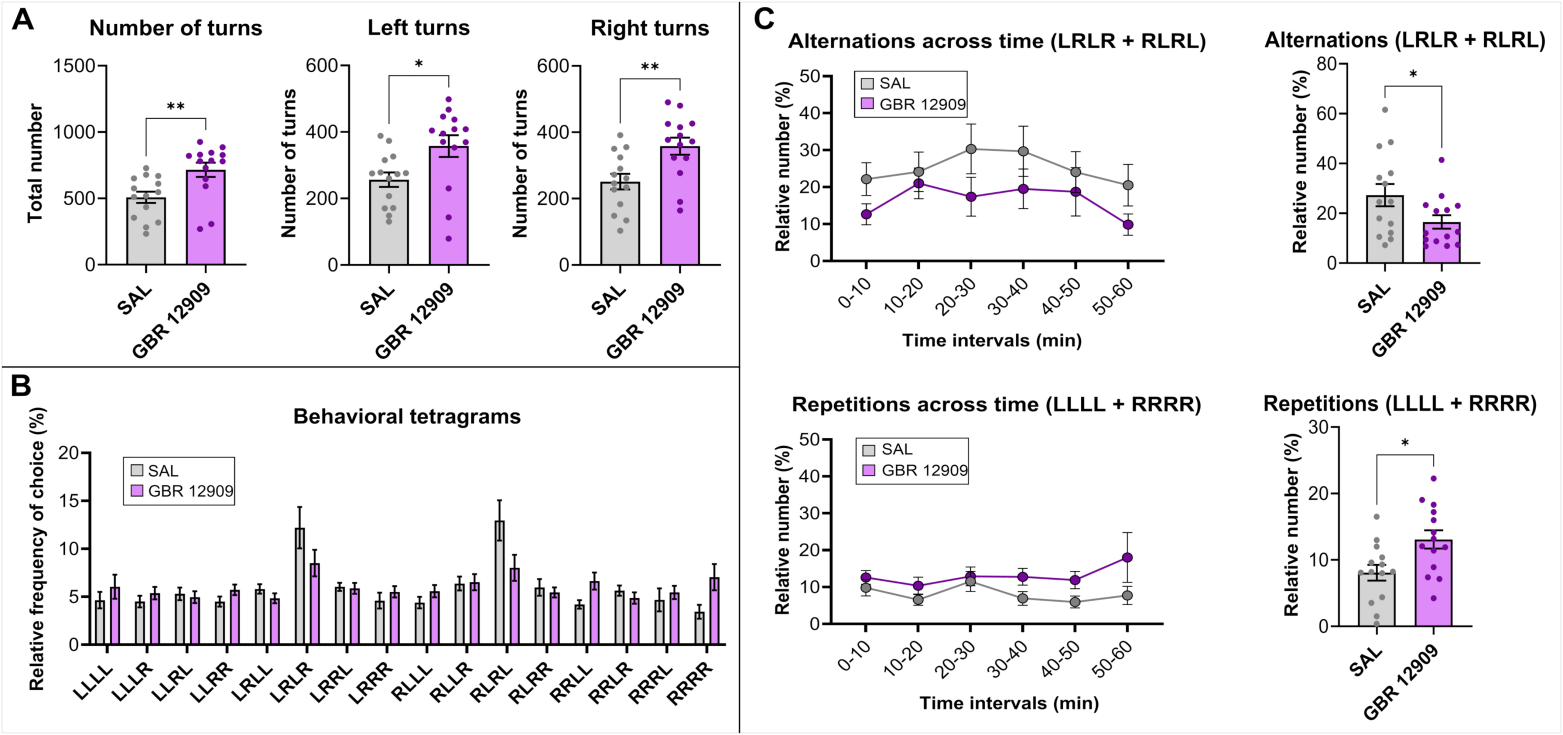
Dopaminergic modulation of GBR 12909 in cognitive functions and working memory in the FMP Y‐Maze test. (A) Overall movement parameters. (B) Frequency of choices. (C) Number of alternations and repetitions assessed as endpoints and across the trial. Temporal analysis of behaviour was calculated as the area under the curve (AUC) for each experimental subject in 5‐min time intervals. Data are expressed as means ± S.E.M and analysed by Student's *t*‐test or two‐way RM‐ANOVA, depending on the experimental approach (*p ≤ 0.05, **p ≤ 0.01, ***p ≤ 0.001, ****p ≤ 0.0001; *n* = 14 per group).

The influence of GBR 12909 on working memory and cognitive flexibility was measured in the FMP Y‐maze test (Figure 4). Regarding the movement patten (Figure 4A), GBR 12909 increased the number of left turns (*t*_*(26)*_ = 2.579, *p* = 0.0159, *d =* 1.0115), right turns (*t*_*(26)*_ = 3.068, *p* = 0.0050, *d* = 1.2033) and, consequently, total number of turns (*t*_*(26)*_ = 3.030, *p* = 0.0055, *d* = 1.1884). Behavioural tetragrams (Figure 4B) revealed that the relative frequency of sequential turn choices showed a tendency to be characterized by an alternated pattern in both groups, a characteristic behaviour across species. RM‐ANOVA revealed significant effect of sequential choices × treatment interaction (*F*_(15, 390)_ = 2.366, *p =* 0.0029, *ηp* ^2^ = 0,0834) and a significant effect of sequential choices (*F*_(2.641, 68.67)_ = 6.777, *p* = 0,0008, *ηp* ^2^ = 0,2067), with no effect of treatment, indicating that the pattern of sequential turn choices differed according to treatment. However, multiple comparisons test revealed no significant differences between the SAL and GBR 12909 groups for any individual tetragram pattern. When the overall behavioural indices were analysed, GBR 12909 group also showed a lower percentage of alternations (*t*_*(26)*_ = 2.068, *p* = 0.0487, *d* = 0.8111), along with increased repetitions (*t*_*(26)*_ = 2.759, *p* = 0.0105, *d* = 1.0821) suggesting impaired working memory and increased repetitive behaviour (Figure 4C). RM‐ANOVA (Figure 4C) yielded no effects of time, treatment or time × treatment for alternations across time. Regarding the repetitions across time, no effects of time or time × treatment effects were reported, but a significant effect of treatment was found (*F*_(1,26)_ = 7611, *p* = 0,0105, *ηp* ^2^ = 0,2264).

## 4 Discussion

Our findings support that pharmacological inhibition of dopamine reuptake with GBR12909 produces a multidomain, context‐dependent behavioural phenotype that encompasses domains associated with BD in adult zebrafish. Here, we assessed multiple behavioural domains and verified marked effects caused by DAT inhibition. Rather than reflecting a single behavioural syndrome, the observed phenotype encompasses changes in homebase occupancy, threat‐related responses, cognitive flexibility and coping strategies. This multidomain profile suggests that acute GBR 12909 induces a context‐dependent behavioural reorganization consistent with dopaminergic dysregulation relevant to BD‐associated behavioural dimensions.

BD is a mental health condition marked by intense shifts in mood, and it often includes symptoms typically seen in anxiety and depressive disorders, with alternation between two distinct phases, manifested in sequential, alternating or mixed patterns: a manic phase, marked by elevated energy and irritability and a depressive phase, characterized by sadness and loss of motivation (Tondo et al. 2017; Jain and Mitra 2025). The dopamine hypothesis suggests that homoeostasis imbalance involving DAT and receptors functions may underlie depressive and manic phases (Ashok et al. 2017). As a selective DAT inhibitor, GBR 12909 reduces the dopamine reuptake by the presynaptic neuron, leading to an excessive accumulation of dopamine in the synaptic cleft (Andersen 1989). Consequently, enhanced dopaminergic neurotransmission alters multiple behavioural and cognitive processes regulated by dopamine (Speranza et al. 2021). The impaired DAT activity contributes to euphoria, irritability, and other symptoms of mania (Young et al. 2010; Canzian et al. 2025). In zebrafish, GBR12909 administration is a pharmacological approach used to induce mania‐ and depression‐like behaviours (Canzian et al. 2024, 2025).

Although BD is a cyclic disorder involving complex genetic, environmental and neurochemical interactions, with symptoms that fluctuate dynamically over time, experimental pharmacological approaches are primarily intended to reproduce specific neurobiological mechanisms and symptom domains associated with the disorder rather than the full spectrum of BD (Scaini et al. 2020; Canzian et al. 2025). In rodents, GBR 12909 elicits hyperactivity in the OFT, as well as anxiety‐like behaviours in the elevated plus maze test, indicating a mania‐like phenotype induced by hypodopaminergic state (Queiroz et al. 2015; Bigot et al. 2022). Accordingly, administration of 15 mg/kg GBR 12909 induces mania‐ and anxiety‐like behaviours in zebrafish, characterized by hyperlocomotion and increased geotaxis in the novel tank test, as well as depression‐like behaviour with increased immobility in the zebrafish tail‐immobilization test (Canzian et al. 2024). However, when exposed in immersion at a concentration of 1‐mg/L GBR 12909 for 20 min, zebrafish showed hypolocomotion in novel tank test, but yet presented an anxiety‐like state, with lower vertical exploration and impaired spatial cognition (Zabegalov et al. 2023). Several factors can be attributed to such differences, including the exposure protocol (i.e., injection vs. immersion) and/or context‐dependent effects of GBR 12909, depending on the behavioural construct measured, also reinforced here in this study. Notably, our findings corroborate that acute GBR 12909 treatment produces a multidomain behavioural phenotype encompassing alterations that reflect mania‐, anxiety‐ and depression‐like responses, probably supporting context‐dependent effects consistent with the involvement of dopaminergic mechanisms in BD.

Our results also showed increased shuttling in LDT and number of turns in the FMP Y‐Maze, as well as anxiety‐like behaviours, supported by the increased scototaxis in the LDT, homebase occupancy in the OFT and stereotypical behaviour with the repetition pattern in the FMP Y‐Maze test. These findings can be associated to dopaminergic overstimulation reported in vertebrate models (Mezzomo et al. 2016; Serchov et al. 2016; Cleal, Fontana, Ranson, et al. 2021; Pretzel et al. 2024; Borba et al. 2025; Fontana et al. 2025). However, dopaminergic activation does not necessarily produce a uniform increase in locomotor output. Instead, it may reorganize behavioural priorities across exploration, vigilance and cognitive control. While lower dopamine levels are often associated with depressive disorders, an excess of dopamine in certain brain regions can induce depressive symptoms, such as reduced motivation, lack of pleasure, and difficulty experiencing emotions, mostly after manic episodes (Pozzi et al. 1994; Canzian et al. 2025; Pardossi et al. 2025). Evidently, the reduced exploratory behaviour and increased immobility found in OFT and SWT, together with the increased pattern of repetitions in FMP Y‐maze are suggestive of anxiety‐, despair‐, defensive‐like behaviours and impaired working memory. These data corroborate that GBR 12909 induces a behavioural profile that engages domains commonly observed during depressive phases of BD, such as reduced motivation, psychomotor changes and cognitive inflexibility (Heikkila and Manzino 1984; Canzian et al. 2022, 2024; Zabegalov et al. 2023).

While previous studies examined the behavioural effects of GBR 12909 in zebrafish behaviour (Zabegalov et al. 2023; Canzian et al. 2024), here we expand this line of investigation by integrating multiple behavioural domains to simultaneously assess mania‐ and depression‐associated responses within the same experimental framework. This integrated approach allows a more comprehensive characterization of how acute DAT blockade mimics distinct behavioural domains relevant to BD. Importantly, our findings also indicate that these behavioural phenotypes are context‐dependent, varying according to the environmental demands and task‐specific parameters, suggesting that DAT dysregulation does not produce a uniform behavioural state, but rather a phenotype modulated by the interaction between neurochemical perturbation and situational context. This aligns with the translational concept of BD as a dynamic disorder rather than fixed trait states (Harrison et al. 2018).

Although anxiety does not represent a main clinical symptom, it is frequently associated with mixed features of BD, characterized by alterations in affective‐like states encompassing anxiety and depressive domains. While the OFT is a behavioural paradigm to measure the spatiotemporal exploratory dynamics of zebrafish (Borba et al. 2025; Mohammed et al. 2025; Pretzel et al. 2024), the LDT assesses the natural preference for darker environments to avoid predation and stressors, using the dark zone as a perceived safe space (Maximino et al. 2011; Magno et al. 2015; Haigis et al. 2022). Together, the OFT and LDT results provide important insights into the behavioural organization induced by distinct pharmacological manipulations (Maximino et al. 2011; Borba et al. 2025). Although GBR 12909 did not increase centre exploration in the OFT, an increased time spent in the homebase was verified, suggesting a spatially restricted exploratory strategy anchored around a preferred location (Borba et al. 2022; Resmim et al. 2023). In the LDT, GBR 12909‐treated fish showed prominent scototaxis supporting anxiety‐like behaviours. These data suggest that DAT inhibition exacerbates defensive behaviours while maintaining a more cautious and spatially constrained exploratory strategy in zebrafish. Importantly, in contrast to previous data showing hyperlocomotion in the novel tank diving test (Canzian et al. 2024), we did not observe significant changes in overall locomotion‐based parameters in the OFT. Nonetheless, the increased shuttling in the LDT could be indicative of increased activity in this task, supporting context‐dependent effects of GBR 12909.

When tested in the SWT, GBR 12909‐treated zebrafish were less active, which may be associated with despair‐like responses, characterized as a sense of hopelessness, reducing the attempts to escape the aversive situation. The respective phenotype can be associated with anhedonia, a characteristic of behavioural despair (Deussing 2006; de Abreu et al. 2020, 2021; Müller et al. 2025). Anhedonia is a symptom of bipolar depression characterized by a decreased ability to experience pleasure, related to the dysregulation of dopaminergic system, since dopamine is responsible for motivation and reward (Höflich et al. 2019). This pattern may also reflect a passive or energy‐conserving coping strategy in an aversive context rather than a simple reduction in motor capacity. Thus, the despair‐like observed may reflect affective and motivational alterations analogous to depressive states found in BD patients.

Dopamine also plays a role in working memory, a cognitive function that temporarily store and manipulate the information necessary to perform specific tasks (Baddeley 1992). In mammalian brain, the main region responsible by this function is the striatum, in which working memory is modulated by the availability of dopamine (Bäckman and Nyberg 2013). In zebrafish, the subpallium or ventral telencephalon is considered homologous to the mammalian striatum, and is likewise implicated in cognitive functions, including working memory (Rink and Wullimann 2001). Here, using the FMP Y‐maze, we found that GBR 12909 increased the number of turns, with higher repeatability of movements and decreased alternated movements, indicating impaired working memory and increased abnormal repetitive behaviours. Temporal analysis also supported a decrease on cognitive flexibility, where zebrafish exposed to GBR 12909 did not increased alternations across time, a typical parameter that reflects cognitive flexibility in this test (Fontana et al. 2025). Prior zebrafish studies employing the same paradigm have demonstrated that D1 receptor antagonism decreases alternation behaviour while increasing repetition (Cleal, Fontana, Ranson, et al. 2021), whereas D1/D5 receptor agonism mitigates the emergence of repetitive response structures following stress exposure (Fontana et al. 2021). This convergence supports the interpretation that GBR 12909‐induced DAT inhibition disrupts striatal‐dependent cognitive processing, yielding behavioural signatures indicative of impaired working memory and reduced cognitive flexibility. Importantly, our findings suggest that the increased repetitive behaviour reflects not merely enhanced locomotion but a shift towards more rigid and stereotyped behavioural organization. Similar patterns of repetitive and rigid motor behaviour have also been reported in zebrafish and rodent models treated with other dopaminergic agents, such as amphetamine and apomorphine, further supporting the interpretation that GBR 12909‐induced stereotypy stems from dopaminergic overstimulation (Ninkovic et al. 2006; Souders et al. 2019; Leggieri et al. 2022). Such alterations show behavioural similarities with the impaired cognitive flexibility, working memory and executive control consistently reported in BD patients, further supporting the translational relevance of this protocol for investigating dopamine‐dependent cognitive dysfunction (Vrabie et al. 2015; Huang et al. 2023).

Although our study observed behavioural alterations in multiple domains relevant to BD, several limitations exist. For example, GBR 12909 administration used here is acute, while BD is a chronic condition with cyclic episodes occurring over months or years (Jain and Mitra 2025). Acute pharmacological induction may not fully reproduce the temporal dynamics, compensatory adaptations, or neuroplastic changes seen in chronic BD. Indeed, a key feature of BD is the spontaneous switching between manic and depressive episodes (Scaini et al. 2020; Jain and Mitra 2025). In this model, manic‐ and depressive‐like behaviours appear after a single treatment and vary according with the paradigm tested, which may not fully mimic the natural oscillation between phases. While GBR 12909 is a selective dopamine reuptake inhibitor, encompassing one molecular mechanism of action (i.e., DAT inhibition), BD may involve interactions between multiple neurotransmitter systems and intricate regulation pathways (Heikkila and Manzino 1984; Kapczinski et al. 2004; Scaini et al. 2020). Thus, a single modulation of dopaminergic activity may oversimplify the neurobiology of BD. Also, changes in dopamine levels in the synaptic cleft may affect differentially dopaminergic D1‐ and D2‐like receptors, requiring further studies to investigate how GBR 12909 influences dopamine‐mediated signalling on specific receptors. Accordingly, future biochemical analyses are necessary to compare dopamine levels between the control and GBR 12909‐administered fish. Acute GBR12909 administration has previously been shown to reduce oxidative stress markers in zebrafish (Canzian et al. 2024), contrasting with the elevated oxidative stress typically reported in BD and in rodent models of mania (Andreazza et al. 2008; Queiroz et al. 2015). This divergence indicates that construct validity of the model warrants further investigation. Finally, it is important to emphasize that previous studies have used a 30‐min period after the injection to evaluate the effects of GBR12909 in zebrafish, which elicited significant effects on locomotion, anxiety‐ and depressive‐related behaviours and oxidative markers (Canzian et al. 2024). Although the pharmacokinetic and pharmacodynamic profiles of GBR 12909 in this aquatic species remain unknown, the effects in primates are known to last between 2 and 5 h depending on the route of administration, and elimination half‐life was estimated at 1–2 days in rodents (Søgaard et al. 1990; Costa et al. 2014). Testing pharmacological approaches commonly used for BD treatment, such as lithium or valproate, would also improve the predictive validity of the model. Despite these limitations, our study reinforces the value of using GBR 12909 in zebrafish to model dopaminergic‐driven behavioural phenotypes relevant to BD across distinct domains.

## 5 Conclusion

In summary, we demonstrate that acute administration to GBR 12909, a selective dopamine reuptake inhibitor, modulates anxiety‐, cognitive‐, affective‐ and exploration‐based functions in adult zebrafish, highlighting the contribution of dopaminergic dysregulation to multiple behavioural domains associated with BD. The present findings should be interpreted as modelling behavioural dimensions relevant to BD rather than reproducing a discrete manic or depressive state. Unlike unidimensional models that isolate either mania or depression, this approach better approximates the multidimensional complexity of the disorder, offering a valid tool for testing mechanistic hypotheses. In fact, the acute GBR 12909 treatment constitutes a multifaceted model that goes beyond a single, isolated mania‐like state. Instead, it induces a multidomain phenotype characterized by context‐dependent activation, increased defensive reactivity and anxiety‐like behaviour, reduced exploratory engagement in aversive settings, and impaired cognitive flexibility. Such profile may better align with a broader framework of dopaminergic dysregulation relevant to BD, including mixed or unstable affective‐like states, rather than a single discrete behavioural syndrome. Our findings also contribute with the utility of zebrafish‐based models as reliable tools for investigating the underlying physiological mechanisms and potential therapeutic interventions for BD and other neuropsychiatric disorders.

## Author Contributions

**Camilla W. Pretzel:** writing – review and editing, writing – original draft, validation, methodology, investigation, formal analysis, data curation, conceptualization. **Hevelyn S. Moraes:** conceptualization, investigation, writing – original draft, methodology, validation, writing – review and editing, formal analysis, data curation. **Mariana L. Müller:** conceptualization, investigation, writing – original draft, methodology, validation, writing – review and editing, formal analysis, data curation. **Kimberly Fontoura:** writing – original draft, methodology, investigation, data curation. **Rossano S. Menezes:** methodology, investigation, data curation. **Julia Canzian:** writing – original draft, writing – review and editing, visualization, formal analysis. **Rodrigo E. Barreto:** writing – review and editing, formal analysis. **Cassio M. Resmim:** writing – original draft, writing – review and editing, visualization, formal analysis. **Barbara D. Fontana:** writing – review and editing, writing – original draft, visualization, formal analysis. **Denis B. Rosemberg:** writing – review and editing, writing – original draft, supervision, resources, project administration, funding acquisition, conceptualization, formal analysis.

## Conflicts of Interest

The authors declare no conflicts of interest.

## Data Availability

Data will be made available on request.
